# The indigenous honey bees of Saudi Arabia (Hymenoptera, Apidae, *Apis mellifera jemenitica* Ruttner): Their natural history and role in beekeeping

**DOI:** 10.3897/zookeys.134.1677

**Published:** 2011-10-06

**Authors:** Abdulaziz S. Alqarni, Mohammed A. Hannan, Ayman A. Owayss, Michael S. Engel

**Affiliations:** 1 Department of Plant Protection, College of Food and Agriculture Sciences, King Saud University, Riyadh 11451, PO Box 2460, KSA; 2 Department of Environmental Sciences, University of Guelph, Guelph, Ontario, Canada N1G 2W1 Current Address: Department of Plant Protection, College of Food and Agriculture Sciences, King Saud University, Riyadh 11451, PO Box 2460, KSA; 3 Division of Entomology, Natural History Museum, and Department of Ecology & Evolutionary Biology, 1501 Crestline Drive – Suite 140, University of Kansas, Lawrence, Kansas 66049-2811, USA; 4 Division of Invertebrate Zoology, American Museum of Natural History, Central Park West at 79th, New York, New York 10024-5192, USA

**Keywords:** Apoidea, Anthophila, Apidae, *Apis mellifera jemenitica*, indigenous honey bee race, beekeeping, morphology, Arabian Peninsula, Saudi Arabia, natural history

## Abstract

*Apis mellifera jemenitica* Ruttner (= *yemenitica auctorum*: *vide* Engel 1999) has been used in apiculture throughout the Arabian Peninsula since at least 2000 BC. Existing literature demonstrates that these populations are well adapted for the harsh extremes of the region. Populations of *Apis mellifera jemenitica* native to Saudi Arabia are far more heat tolerant than the standard races often imported from Europe. Central Saudi Arabia has the highest summer temperatures for the Arabian Peninsula, and it is in this region where only *Apis mellifera jemenitica* survives, while other subspecies fail to persist. The indigenous race of Saudi Arabia differs from other subspecies in the region in some morphological, biological, and behavioral characteristics. Further taxonomic investigation, as well as molecular studies, is needed in order to confirm whether the Saudi indigenous bee populations represent a race distinct from *Apis mellifera jemenitica*, or merely an ecotype of this subspecies.

## Introduction

It is somewhat ironic that most domesticated animals are often overlooked for basic research into their natural history and systematics. This is certainly true for honey bees (*Apis* Linnaeus), where despite millennia of domestication and centuries of intensive biological research the systematics of the species and infraspecific varieties, along with the critical biological attributes of these populations, remains confused (e.g., *vide* Engel 1999 for an overview of the complicated taxonomic history of species of *Apis*). Naturally, we know more about honey bee biology than any other group of Apoidea but this voluminous literature exists despite the existence of significant challenges to the basic systematics, taxonomy, identification, and population variation of the more variable species such as *Apis cerana* Fabricius and *Apis mellifera* Linnaeus (although significant strides are being made; e.g., Hepburn and Radloff 1998, 2011; Radloff et al. 2010, 2011; Meixner et al. 2011).

Perhaps the region most needing investigation today is the fauna of the Arabian Peninsula, along with its neighboring regions. Herein we provide a brief overview of the history of beekeeping in Saudi Arabia, research on the indigenous bee populations, and the available natural history information for these unique honey bees. It is hoped that such a review, highlighting the unique attributes of these honey bees, will further the efforts of systematic, taxonomic, ecological, and apicultural research in the region and thereby provide a stronger foundation to the melittological and apicultural communities in Saudi Arabia.

## History of Apis and Arabian beekeeping

Today’s honey bees diverged from their primitive relatives among the Electrapini, or stem-group Electrapini+Apini, perhaps as long ago as the Eocene-Oligocene transition (ca. 35 Ma) or latest Eocene (ca. 40 Ma) (Engel 1998, 2001a, 2001b, 2006; Engel et al. 2009). The earliest records of fossils definitively attributable to the genus *Apis* are from the Early Oligocene of Europe, about 35 Ma (Zeuner and Manning 1976; Engel 1998, 1999). Indeed, these earliest honey bees, such as *Apis henshawi* Cockerell and *Apis vetusta* Engel superficially resemble to a large degree modern workers of *Apis mellifera* L. (Engel 1998; Kotthoff et al. 2011). During the Oligocene and particularly the Miocene, honey bees exhibited significant morphological variability within populations, extended their distributions into Asia, Africa, and even the New World (Engel et al. 2009), and diversified into the principal lineages (i.e., subgenera) we recognize among modern species of *Apis* (Engel 2006; Engel et al. 2009; Kotthoff et al. 2011). By the origin of modern humans, honey bee diversification had already taken place and their varieties had well established populations and it was not long before early groups of *Homo sapiens* L. began to exploit the resources species of *Apis* had been producing for millions of years.

**Figures 1–4.**
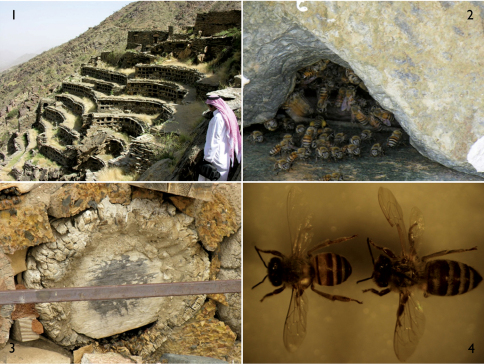
Bees and beekeeping in Saudi Arabia. **1** A historical apiary with traditional hives of Saudi *Apis mellifera jemenitica* Ruttner maintained over 500 years by the same family in Taif (there are many such apiaries in the area, with beekeepers maintaining these as a family tradition over numerous generations; honey from such apiaries is much costlier than those managed in Langstroth hives) **2** Entrance to a hive of *Apis mellifera jemenitica* in Taif **3** A traditional log hive of *Apis mellifera jemenitica* in Taif **4** Photograph showing size and other morphological differences between *Apis mellifera jemenitica* and *Apis mellifera carnica* Pollmann.

While beekeeping is at least 4500 years old and honey hunting (i.e., relying solely on feral colonies rather than domesticated hives and therefore not true “beekeeping”) even more ancient, dating to at least 6000 BC (Galton 1971; Dams 1978; Crane 1983, 1990, 1999), the record from Arabia is not quite as extensive. In ancient Egypt beekeeping was practiced as early as 2500 BC (Crane and Graham 1985; Crane 1999) and in Greece the practice was diversified by at least the Minoan period (Crane 1999). During this early phase of beekeeping, bees were kept in earthen pots, cylinders, or logs which were placed horizontally and either hung or stacked. The history of beekeeping in the Arabian Peninsula dates back to ca. 2000 BC. An earthen painting found in Iraq depicts honey as a remedy for diseases (Crane 1983), and writers of antiquity often mentioned the riches of the beekeeping industry in the region. For example, Blinos (79 AD) noted that, “Arabia Felix wealth outperformed the whole world, as its lands had perfumed jungles, gold mines, irrigating water and produced a lot of honey and wax” (Tarcīci 1968). Similarly, Strabo (64 BC–24 AD) considered honey as one of the prominent products of “Arabia Felix”, indicating in his *Geographica*, “the far western parts, towards Ethiopia, were irrigated by summer rainfall and cultivated twice a year, and honey was one of its numerous yields and was enormously abundant” (Jones 1930 [Strabo Book 16-3]). Clusters of the oldest apiaries in the region can be found in Taif, southwestern Saudi Arabia. According to apiary owners these were built in the mountain around 500 years ago (Figs 1–2). Cylinder log hives were kept in elongate cells of the rock face so as to protect them from possible attacks by rival tribes.

Early Arabic literature reveals that Arabs in the Arabian Peninsula recognized and kept bees for honey production. They called bee hives “kawarah”, which means a habitation made of stalks, mud, or a wooden cavity. They also named apiaries as “masane’a”, meaning “factories”, which were kept at isolated sites away from human habitation (Al-Zabīdī 1888/1889). The Arabs also recognized the individual castes of the colony such as the queen (termed “the prince”) and drones (“the biggest and darkest ones that stay in the nest, eat honey, and not produce it”: Al-Zabīdī 1888/1889). In addition, they made detailed descriptions of swarming behavior and the various developmental stages such as eggs and larvae (Al-Zabīdī 1888/1889). Furthermore, they recognized bee plants such as *Schanginia hortensis* (Forssk. ex J.F. Gmel.) Moq. [Amaranthaceae], *Blepharis ciliaris* (L.) B.L. Burtt. [Acanthaceae], *Lavandula* spp. [Lamiaceae], *Ziziphus* spp. [Rhamnaceae], *Acacia asak* (Forssk.) Willd. [Fabaceae (a.k.a., Leguminosae)], *Acacia senegal* (L.) Willd. [Fabaceae (a.k.a., Leguminosae)], and *Anisotes trisulcus* (Forssk.) Nees [Acanthaceae]. Faith and interest in honey and honey bees increased greatly in the Arabian Peninsula after one chapter in The Holy Quran was entitled “Al-Nahl – The Bees”, in which honey was mentioned as “a curative for mankind”.

The modern era of beekeeping was initiated over 150 years ago. The development of the Langstroth bee hive in 1851 boosted the beekeeping industry (Langstroth 1853), and since that time significant advances have continued to be made in methods of boxing of bees, creating apiaries, observing hives, treating pathologies, &c. Some of these advances included methods of queen rearing which were restricted to a single type between 1850–1890 (Pellett 1938) but have explosively diversified over the last century, particularly with the advent of successful means of artificial insemination (Watson 1928; Laidlaw 1944; Mackensen and Roberts 1948), thereby permitting the more rapid and effective development of novel strains, e.g., genetic lines developed bees high brood production, greater honey yields, selectivity for sugar solutions, resistance to viral diseases, etc. (e.g., Cale and Gowen 1956; Nye and Mackensen 1968; Kulinčević and Rothenbuhler 1975). Naturally, these and many other advances are employed widely in Saudi Arabia, although many traditional and often ancient beekeeping practices are simultaneously in widespread use (e.g., Figs 1–3).

## Races of honey bees (Apis mellifera)

Engel (1999) reviewed and reported the presence of 28 races of *Apis mellifera*, and 10 valid geographical races in Africa, although Meixner et al. (2011) recently have added an additional subspecies to this fauna. Table 1 provides an updated list of races of *Apis mellifera*, including the area in which they occur. Not all are considered valid by all authors (e.g., Engel 2006). As has been noted by several authors for honey bees in general, the various subspecies are not discrete units and the utility of a subspecific classification has been challenged (e.g., Hepburn 2000; Hepburn and Radloff 1998, 2002, 2011; Radloff et al. 2010). It is well beyond the scope of the present review to tackle this issue but it must be noted and seriously addressed by the apidological research community.

**Table 1.** Summary of subspecies of *Apis mellifera* widely used in the apidological and apicultural literature and industry, arranged loosely by Ruttner’s (1988) gross geographical areas (not all are considered valid biologically or taxonomically; e.g., Engel 1999, 2006).

Western Mediterranean
*Apis mellifera iberiensis* Engel
*Apis mellifera intermissa* Maa
= *major* Ruttner
*Apis mellifera mellifera* Linnaeus
*Apis mellifera ruttneri* Sheppard et al.
*Apis mellifera sahariensis* Baldensperger
Irano-Ponto-Caspian East Mediterraean / Central Asia
*Apis mellifera adami* Ruttner
*Apis mellifera anatoliaca* Maa
*Apis mellifera caucasia* Pollmann
*Apis mellifera cypria* Pollmann
*Apis mellifera meda* Skorikov
*Apis mellifera pomonella* Sheppard & Meixner
*Apis mellifera remipes* Gerstaecker
= *armeniaca* Skorikov
*Apis mellifera sossimai* Engel
*Apis mellifera syriaca* Buttel-Reepen
*Apis mellifera taurica* Alpatov
Central Mediterranean-Southeastern Europe
*Apis mellifera carnica* Pollmann
*Apis mellifera carpatica* Barac
*Apis mellifera cecropia* Kiesenwetter
*Apis mellifera ligustica* Spinola
*Apis mellifera macedonica* Ruttner
*Apis mellifera siciliana* Grassi
= *sicula* Montagano
African-Arabian
*Apis mellifera adansonii* Latreille
*Apis mellifera bandasii* Amssalu
*Apis mellifera capensis* Eschscholtz
*Apis mellifera lamarckii* Cockerell
*Apis mellifera litorea* Smith
*Apis mellifera monticola* Smith
*Apis mellifera jemenitica* Ruttner
= *nubica* Ruttner
*Apis mellifera scutellata* Lepeletier de Saint Fargeau
*Apis mellifera simensis* Meixner et al.
*Apis mellifera sudanensis* El-Sarrag et al.
*Apis mellifera unicolor* Latreille
*Apis mellifera woyigambella* Amssalu

## Saudi beekeeping

Beekeeping in Saudi Arabia is a growing industry. The estimated numbers of beekeepers and bee hives are 4000 and 700,000, respectively, and they produce collectively about 3500 tons of honey per year, or about 26% of the required demand. As a result, approximately 10,000 tons of honey is imported annually from Europe, Iran, Turkey, Australia, the United States, and to a lesser extent from other countries. Accordingly, during the last couple of decades researchers have paid special attention to various aspects of beekeeping in the region, including critical investigations into honey bee races, the biology of native populations, queen rearing, bee pests and diseases, climatic impacts, as well as bee economics (e.g., El-Sarrag 1993; Al-Qahtani 1997; Al Ghamdi 1990, 2002, 2005, 2006; Alqarni 1995, 2006a, 2006b, 2010; Alqarni and Al-Atawi 2008; Alshehri 1999; Al-Kahtani 2003).

With an area of more than 2 million km^2^, the climate of Saudi Arabia is relatively wide ranging, with temperature and rainfall representing the key factors influencing beekeeping activities. Temperatures start to increase in March–April and both are hot months but considered as merely transitional, whereas the period from May–August is extremely hot. Maximum temperatures during July are more than 40° C, and temperatures do not start declining until September–October, although both are still quite hot months. Temperatures range from moderate to very cold until February, with January being the coldest month of the year (sometimes around -7° C in the North). Annual rainfall ranges from a few millimeters in the Rubu–Alkhali desert to 600 mm in the mountainous areas of the Southwest. These variations in temperature force beekeepers to search for different foraging areas for their bees each year.

**Figure 5.**
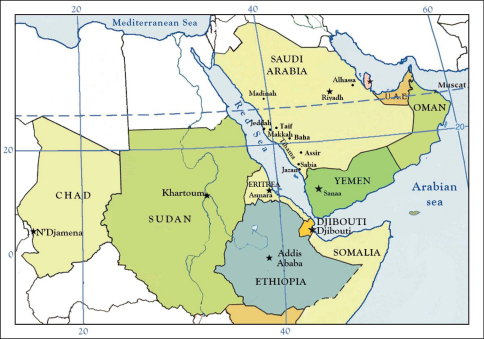
Distribution of *Apis mellifera jemenitica* Ruttner in the Arabian Peninsula and northeastern Africa.

Taif, Baha, and Assir (mountainous regions) in the Southwest are the most suitable areas for keeping bees in Saudi Arabia (Fig. 5). These areas comprise 762,474 acres of forests with an altitude of 900–3700 m (Abu-Hassan et al. 1994). The most common species of trees are *Acacia* spp. [Fabaceae (a.k.a., Leguminosae)], *Olea europaea* subsp. *cuspidata* (Wall. & G.Don) Cif. [Oleaceae; often under the synonym *Olea chrysophylla* Lam.], *Juniperus procera* Hochst. ex Endl. [Cupressaceae], *Hyphaene thebaica* (L.) Mart. [Arecaceae], and *Ziziphus spina-christi* (L.) Willd. [Rhamnaceae] (Al-Owdat et al. 1985). Temperatures in summer and winter in these regions ranges from 20°–28° C and 9°–14° C, respectively. During winter, beekeepers take their bees down to Tihama, a warm coastal region harboring several rich pollen plants that help beekeepers to increase the number of their hives through uncontrolled swarming. Since beekeepers follow traditional beekeeping methods, swarming is allowed to occur freely and more than one swarm normally leaves the hive. Unfilled traditional hives (hollow logs) marked with beeswax are distributed in the vicinity to attract swarms (Fig. 3). Other swarms are captured from trees and placed in empty hives. Most beekeepers in the Southwest perform traditional beekeeping methods, whereas Langstroth hives are used in other parts of the country. Most beekeepers perform migratory beekeeping to avoid severe weather and food deficiency. In the central region, wild nectar and pollen plants such as *Astragalus spinosus* (Forssk.) Muschl. [Fabaceae (a.k.a., Leguminosae)], *Horwoodia dicksoniae* Turrill [Brassicaceae], *Anisosciadium isosciadium* Bornm. [Apiaceae], *Citrullus colocynthis* (L.) Schrad. [Cucurbitaceae], *Achillea fragrantissima* (Forssk.) Sch.Bip. [Asteraceae (a.k.a., Compositae)], *Capparis spinosa* L. [Capparaceae], *Acacia* spp. [Fabaceae (a.k.a., Leguminosae)], and *Ziziphus spina-christi* [Rhamnaceae] are available for bees after the rainy season, in addition to cultivated alfalfa, eucalyptus, sunflower, date palm, and some fruit trees (Alghoson 2004; Aloraydh and Alfarraj 1994). Beekeepers follow the flowering of these plants within an area of 400–500 km in diameter in the central region of the country. Some beekeepers move their bees across the country from South to West, to the Center, or to the North and vice versa. In general, the main honey plants in the country are *Acacia* spp. [Fabaceae (a.k.a., Leguminosae)] and *Ziziphus spina-christi* [Rhamnaceae], both being found wild in all regions of Saudi Arabia. Their flowering seasons start during June and August of each year, respectively, depending on rainfall. Approximately 70% of the bees kept in Saudi Arabia are native populations of *Apis mellifera jemenitica*, with the remainder being Carniolan (*Apis mellifera carnica* Pollmann) or Egyptian-Carniolan hybrids (Fig. 4).

## Distribution, morphology, and biology of the native honey bees of Saudi Arabia (Apis mellifera jemenitica)

*Apis mellifera jemenitica* has a wide distribution (4500 km from East to West) in tropical Africa, and in the hot desert of the Arabian Peninsula (Fig. 5), having been recorded from Chad, Ethiopia, Somalia, Sudan, Oman, Yemen, and Saudi Arabia. It is found in the areas of highest seasonal temperature as well as the zone of lowest and most irregular rainfall, regions where other honey bee races have not been able to persist (Ruttner 1976; Fletcher 1978; Radloff and Hapburn 1997; Amssalu et al. 2004; Taher et al. 2009).

Although the native Saudi honey bee is recognized as *Apis mellifera jemenitica*, it has some significant morphological and biological differences from its conspecifics, as well as some other populations of this same race in Africa (Alqarni 1995, 2006, 2010; Al Ghamdi 1990, 2002, 2005, 2006). Ruttner (1988) placed *Apis mellifera jemenitica* (with the unjustified change in spelling to *yemenitica*: *vide* Engel 1999) under the ‘Tropical African region’ as it was found between Chad and Sudan (Fig. 5). He mentioned the subspecies to be one of the major branches of *Apis mellifera*, whose morphology and ecology were extreme. Alpatov (1935) and Guiglia (1964, 1968) had earlier recorded bees from Yemen but owing to a lack of morphometric data confirmation of subspecific status was not possible. In 1970, H. Peters collected in northern Yemen what he presumed to be *Apis cerana indica* Fabricius but Ruttner (1976) correctly identified these bees as small individuals of *Apis mellifera*, resembling otherwise sub-Saharan forms. Ruttner (1988) also noted that *Apis mellifera jemenitica* nearly overlaps *Apis cerana* Fabricius in size and setal length. Ruttner (1976) classified the race found in Sudan as *Apis mellifera nubica* Rutter, later discovering that the same or similar form was also present in Yemen, Somalia, and Chad. As a result, he extended *Apis mellifera jemenitica* into these regions and considered *Apis mellifera nubica* to be a junior synonym of the former (Ruttner, 1988). In total Ruttner (1988) sampled localities across Saudi Arabia [Southwest (Sabia), middle (Riyadh), and East (Alhasaa)], Oman (Dutton et al. 1981), Yemen, Somalia, Sudan (Ruttner 1976, Rashad and El-Sarrag 1983, 1984), and Chad (Gadbin et al. 1979), and representing approximately the same latitude.

Rashad and El-Sarrag (1983, 1984) and El-Sarrag et al. (1992), after surveying 50 localities across eight Sudanese governorates, documented what they considered to be two subspecies from Sudan – *Apis mellifera sudanensis* Sarrag et al. (from most major sections of the country) and *Apis mellifera jemenitica* (listed at times as *Apis mellifera nubica*, along the southern borders). The Sudanese bees were a little larger than the Saudi populations, and those from Somalia mostly resemble those of Sudan. Across all of the morphometric characteristics it was found that the populations from Saudi Arabia were the smallest, with the bees from Chad being perhaps most similar to *Apis mellifera jemenitica* (Gadbin et al. 1979; Gadbin 1980). However, Ruttner (1988) suggested the contrary, believing *Apis mellifera jemenitica* to be more closely allied to those populations from Somalia and Sudan.

Ruttner (1988) noted that the Saudi honey bee samples were clearly distinct (i.e., smaller, more slender, shorter setae, and more yellow in coloration), while adding that there was more homogeneity between Yemeni and Sudanese bees. This led him to be the first to consider the Saudi populations as a distinct ecotype of *Apis mellifera jemenitica*, and the Sudanese population another, less distinctive, ecotype. Naturally, given that increased scrutiny has continued to subdivide the Ethiopian and Sudanese populations into separate subspecies, it must be wondered if the same is not valid for the native Saudi honey bees (*vide* Conclusions, *infra*).

Subsequent studies by Alqarni (1995) demonstrated significant differences in several morphological characters between Saudi *Apis mellifera jemenitica*, the standard ‘Carniolan race’ (*Apis mellifera carnica*), and their F1 hybrid cross (the ‘Arabian-Carnica’ honey bees) (Table 2). The native Saudi honey bees are noticeably smaller and could perhaps be classified as a race distinct from *Apis mellifera jemenitica* (if employing the same standards applied elsewhere for honey bee subspecies; e.g., Sheppard and Meixner 2003; Amssalu 2002, 2008; Sheppard et al. 1997; Amssalu et al. 2004; Meixner et al. 2011), but at least represents a noteworthy ecotype. Indeed, across a variety of biological and behavioral characters (e.g., worker brood, honey and pollen stored, foraging activity and time), the native Saudi populations of *Apis mellifera jemenitica* performed better than *Apis mellifera carnica* or F1 hybrids of these subspecies (e.g., Tables 3–6). The Saudi populations of *Apis mellifera jemenitica* appear better suited than other races for survival and activity (e.g., foraging) in the extremes of the harsh Saudi environment. Alqarni (1995) also considered that the geographical proximity of the native Saudi bees (in the Abha Region) to Yemeni bees (*Apis mellifera jemenitica* s.str.) lead to a natural intermingling between these populations. When comparing the mean values of various morphometric characters of native Saudi bees with the Yemeni populations, Alqarni (1995) noted significant differences among five characters, i.e., length and width of the forewing, width of the metabasitarsus, total length of the third and fourth metasomal terga, and the degree of yellow coloration on the third metasomal tergum. Less significant differences were found in other standard characters, e.g., length of proboscis, cubital cell index, number of hamuli on the hind wing, and slenderness of the metasoma.

**Table 2.** Range and mean values (mm) of some morphometric characters of workers of native Saudi (*Apis mellifera jemenitica* Ruttner) and carniolan races (*Apis mellifera carnica* Pollmann), and their F1 hybrid (100 workers/sample/race).

Morphometric character	*Apis mellifera jemenitica*		*Apis mellifera carnica*		First hybrid	
	Range	Mean	Range	Mean	Range	Mean
Flagellum length	2.32–2.64	2.47±0.01	2.58–2.84	2.70±0.01	2.52–2.77	2.64±0.01
Proboscis length	4.84–5.74	5.31±0.02	5.17–6.32	6.06±0.01	4.52–6.06	5.65±0.02
Forewing length	7.55–9.39	8.07±0.02	8.45–9.22	8.86±0.02	7.93–9-03	8.49±0.03
Forewing width	2.77–3.23	3.01±0.01	3.03–3.40	3.24±0.01	2.90–3.29	3.14±0.01
Forewing cubital index	1.43–2.67	2.10±0.03	1.75–3.33	2.81±0.04	1.75–3.00	2.39±0.04
No. of hamuli on hind wing	18.0–32.0	22.7±0.28	18.0–26.0	21.2±0.18	18.0–29.0	22.65±0.23
Metabasitarsus length	1.94–2.26	2.12±0.01	2.26–2.58	2.44±0.01	1.99–2.39	2.25±0.01
Metabasitarsus width	0.97–1.16	1.08±0.00	1.09–1.23	1.19±0.00	0.97–1.23	1.13±0.01
No. of setal rows on metabasitarsus	10.0–12.0	11.2±0.05	11.0–13.0	11.7±0.07	10.0–12.0	11.1±0.04
Length of metasomal terga III & IV	3.42–3.99	3.75±0.01	3.48–4.26	3.86±0.02	3.48–4.06	3.79±0.02
Yellow color (%) of the metasoma	40.0–75.9	59.0±0.01	0.0–55.0	6.0±0.01	0.0–56.7	29.0±0.02
Metasomal slenderness	79.2–90.7	83.78±0.00	81.3–95.6	85.69±0.01	82.2–97.5	87.5±0.00

**Table 3.** Monthly mean values of some biological characters of native Saudi (*Apis mellifera jemenitica* Ruttner) and carniolan (*Apis mellifera carnica* Pollmann) colonies and their F1 hybrid during a single year (July 1992–June 1993). Different letters in the same row indicate significant differences.

Biological character	Monthly Mean Values of Colonies		
	*Apis mellifera jemenitica*	*Apis mellifera carnica*	first hybrid
Sealed worker brood areas (in^2^)	415.22±54.85 a	267.27±46.74 b	451.48±71.82 a
Sealed drone brood areas (in^2^)	18.95±5.17 a	7.77±2.36 b	7.90±2.24 b
Stored honey (kg)	2.33±0.20 a	2.83±0.30 a	2.76±0.29 a
Stored pollen areas (in^2^)	58.14±8.36 a	29.68±5.42 b	25.69±5.46 b
No. of queen cells	2.94±1.45 a	1.55±0.49 a	3.6±1.95 a
No. of wax cups	10.81±8.21 a	6.93±3.43 a	13.41±3.88 a

**Table 4.** Levels of some behavioral characters of sealed worker brood of native Saudi (*Apis mellifera* *jemenitica* Ruttner) and carniolan (*Apis mellifera carnica* Pollmann) colonies and their F1 hybrid during one year (July 1992–June 1993).

**Character**	**Season**	**Levels given to colonies (1 degree/5 degrees)**		
		*Apis mellifera jemenitica*	*Apis mellifera carnica*	first hybrid
Hardness	WinterSummer% Variance	1.712.6321.2+	1.251.6212.9+	1.632.6123.1+
Range	WinterSummer% Variance	2.442.888.3+	1.723.2030.1+	2.103.4023.2+
Arrangement	WinterSummer% Variance	3.403.301.5-	2.803.206.7+	3.134.2014.6+
Grading	WinterSummer% Variance	3.503.204.5-	2.903.306.5+	3.203.200.0
Total	WinterSummer	11.0512.01	8.6711.32	10.0613.41
General mean	“20 degrees”	11.53	10.00	11.74
Percentage		57.65	49.98	58.68

**Table 5.** Worker brood, honey, and pollen stored by native Saudi (*Apis mellifera jemenitica* Ruttner) and carniolan (*Apis mellifera carnica* Pollmann) colonies and their F1 hybrid during winter (Early December 1992–Late February 1993).

**Biological Character**	**Month**	**Activities of Colonies**					
		*Apis mellifera jemenitica*	variance	*Apis mellifera carnica*	variance	first hybrid	variance
Worker brood (in^2^)	Dec.Feb.	189.8079.00	-58.4%	61.00122.50	+50.2%	161.00112.00	-30.4%
Stored honey (kg)	Dec.Feb.	1.641.14	-30.5%	2.750.65	-76.5%	3.171.01	-68.1%
Pollen (in^2^)	Dec.Feb.	53.3035.30	-33.8%	15.5012.80	-17.4%	16.306.80	-58.3%

**Table 6.** Flight activity of native Saudi (*Apis mellifera jemenitica* Ruttner) and carniolan (*Apis mellifera carnica* Pollmann) colonies and their F1 hybrid during Early February to Late May (1992/1993).

**Observation**	**Time of Activity for Colonies**		
	*Apis mellifera jemenitica*	*Apis mellifera carnica*	first hybrid
Time of 1^st^ worker flight	5:55	5:56	5:53
Time of last worker return	18:09	17:58	18:13
**Time of sunrise**	6:02	**Time of sunset**	18:03
**Sunrise temperature**	14.5°C	**Sunset temperature**	23.5°C

Overall, Saudi *Apis mellifera jemenitica* appears well adapted to the unique climatic conditions and their variations in the Kingdom of Saudi Arabia. For example, their rate of new queen development and foraging activities during the hot summer were significantly higher than those of other races (Alqarni 2006a, 2010). *Apis mellifera jemenitica* is also the smallest of its kind in the area but the biological significance of this size differences is presently not understood. All of the presently available data taken into consideration, it appears as though the native Saudi honey bees are an ecotype of the Yemeni race (i.e., *Apis mellifera jemenitica* s.str.) and one that is ideal for the particular climatic regimes of the country.

Interestingly, Ruttner (1976) observed significant morphological differences among workers of the same colony in his samples across Yemeni, Sudanese, and Chadian populations, leaving him unable to explain these distinctions across the same latitude. This was also noted by El-Sarrag for honey bees sampled across eight governorates in Yemen (*vide* Alqarni 1995), which obviously did not belong to the pure Yemeni race (*Apis mellifera jemenitica* s.str.). Migratory beekeeping and the annual import of thousands of colonies of different hybrids likely explains these observations, as the probability of pure Yemeni bees crossing with other races and hybrids must be great. Genetic studies are needed in order to determine the true frequency and effects of such hybridization in these regions. A great risk of crossing between races in different parts of Saudi Arabia is to be expected. As already mentioned, regions such as Assir, Baha, Taif, Tihama, and other parts of the country import numerous colonies from abroad. Importation is presently uncontrolled and crosses between colonies are to be expected, resulting in mixed drones mating with virgin queens, something which has already been observed among Carniolan bees imported from Egypt.

## Conclusion

Extensive biological, ecological, and systematic studies remain to be undertaken on honey bee populations across the Arabian Peninsula and it is hoped that this brief review will direct researchers to the limited available publications and data. Certainly a question needing further investigation is to origin and distinctiveness of the native Saudi honey bee populations and whether they are sufficiently different to warrant separate subspecific status relative to “true” *Apis mellifera jemenitica* populations in Yemen. In particular, greater and finer geographic sampling of morphometric data is needed (as has been applied to populations and ecotypes of *Apis cerana*: Radloff et al. 2010), alongside extensive DNA sequence analyses. Detailed genomic study of the samples from these regions along with such finer geographic sampling of morphometric variables will solve eventually the challenge surrounding the appropriate classification of the indigenous Saudi honey bees. Given that these subspecies and ecotypes can have profoundly different biological characters, rendering them able (or not able!) to survive in the harsh extremes of the Saudi environments, these seemingly basic questions can have a direct impact on beekeeping efforts. All of these basic investigations into Saudi bee biology will greatly aid national initiatives to strengthen the beekeeping industry and economy of the region, demonstrating once more a direct link between largely systematic research and human agriculture, health, cultural practices, and economic stability. For the moment we maintain a conservative position in regard to the status of our Saudi populations and seek to garner larger and more diverse data sources, much as has been done for near neighbors in Africa and elsewhere in Asia. The Arabian Peninsula represents a significantly open field of inquiry for apidological study.
